# Supplementary figures and images for: Metabolic risk factors and incident advanced liver disease in non-alcoholic fatty liver disease (NAFLD): A systematic review and meta-analysis of population-based observational studies
Source: PLoS Med. 2020 Apr 30;17(4):e1003100. doi: 10.1371/journal.pmed.1003100 (PMC7192386; doi:10.1371/journal.pmed.1003100)

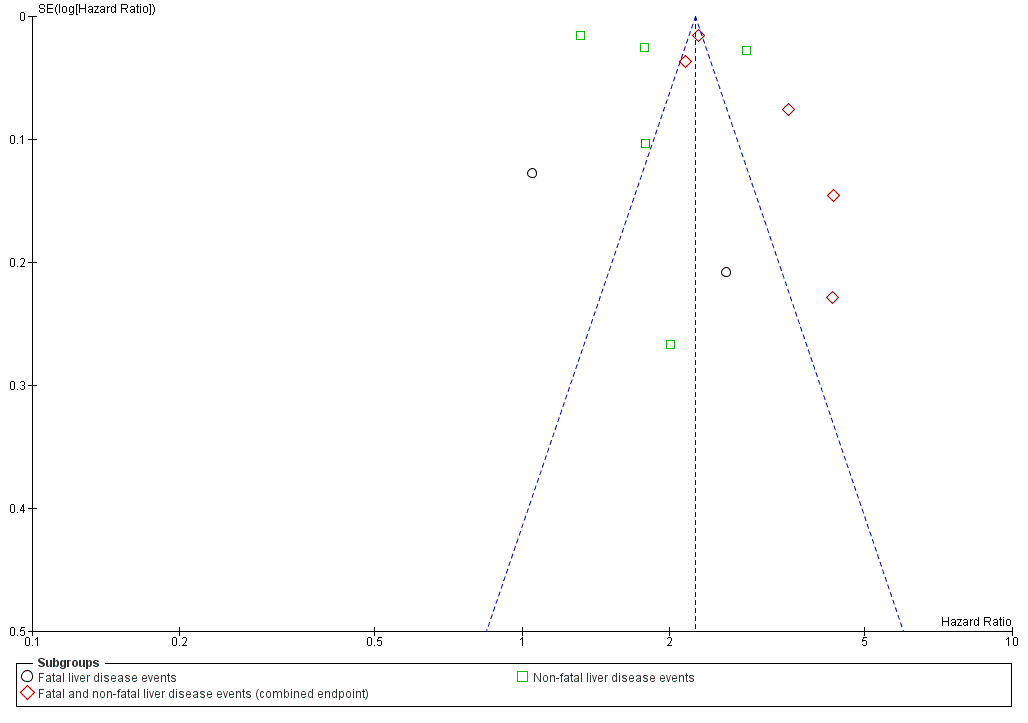

Supplement: S1 Fig — (TIF) [file pmed.1003100.s001.tif]

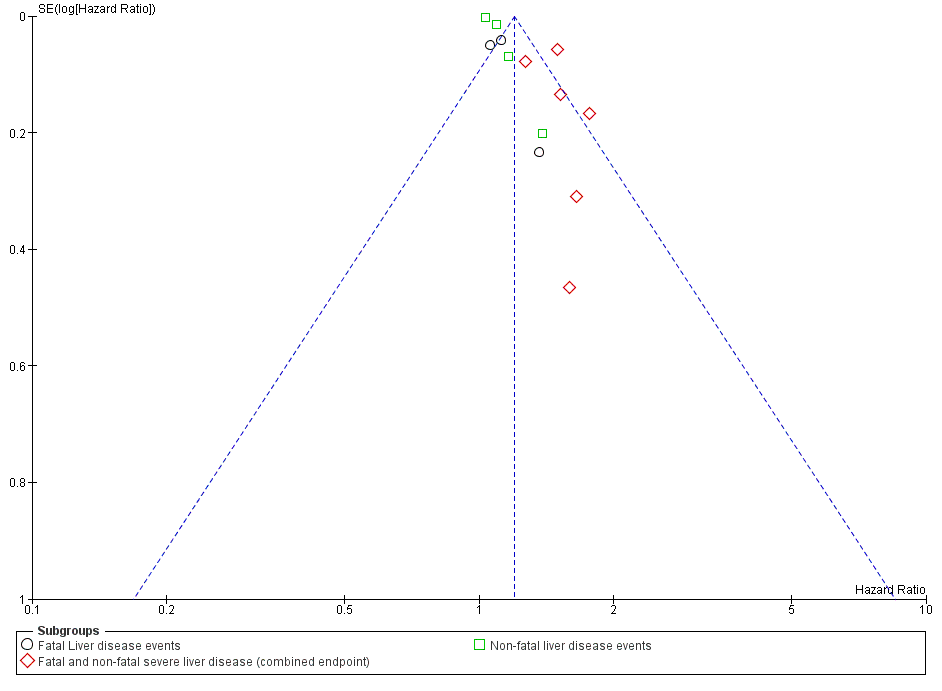

Supplement: S2 Fig — (TIF) [file pmed.1003100.s002.tif]
