# Supplementary material for: Metabolic risk factors and incident advanced liver disease in non-alcoholic fatty liver disease (NAFLD): A systematic review and meta-analysis of population-based observational studies
Source: PLoS Med. 2020 Apr 30;17(4):e1003100. doi: 10.1371/journal.pmed.1003100 (PMC7192386; doi:10.1371/journal.pmed.1003100)
Supplement: S2 Table — (DOCX) [file pmed.1003100.s004.docx]

S2 Table: Medline search strategy

| 1 exp fatty liver/ |
| --- |
| 2 exp non-alcoholic fatty liver disease/ |
| 3 (liver and (fatty or steato*)).ti,ab. |
| 4 NAFLD.ti,ab. |
| 5 1 or 2 or 3 or 4 |
| 6 metabolic syndrome/ |
| 7 (metabolic adj1 (syndrom* or profile)).ti,ab. |
| 8 (syndrome adj1 (insulin resistance or visceral obesity)).ti,ab. |
| 9 waist circumference/ |
| 10 (waist adj (circumference or size)).ti,ab. |
| 11 body mass index/ |
| 12 (((body mass or quetelet) adj index) or bmi).ti,ab. |
| 13 triglycerides/bl |
| 14 hypertriglyceridemia/ |
| 15 (hypertriglycerid?emi* or ((raise* or high or elevat* or increase*) adj2 triglycerid*)).ti,ab. |
| 16 exp hypoalphalipoproteinemias/ |
| 17 lipoproteins, hdl/bl |
| 18 (hypoalphalipoprotein?emi* or ((hdl or ((high density or high-density or alpha or heavy) adj1 lipoprotein*)) adj2 (low or lower* or hypo or deficien*))).ti,ab. |
| 19 Diabetes Mellitus, Type 2/ |
| 20 (diabet* adj2 (type 2 or type2 or typeii or type two)).ti,ab. |
| 21 (dm2 or t2d*).ti,ab. |
| 22 (diabet* adj2 (noninsulin or non insulin or slow-onset or slow onset or adult-onset or adult onset)).ti,ab. |
| 23 exp hypertension/ |
| 24 (hypertens* or high blood pressure*).ti,ab. |
| 25 or/6-24 |
| 26 5 and 25 |
| 27 NASH.mp. |
| 28 steatohepatitis.mp. |
| 29 liver fibrosis.mp. |
| 30 liver cirrhosis/ |
| 31 exp fatty liver/ or non-alcoholic fatty liver disease/ |
| 32 Carcinoma, hepatocellular/ |
| 33 (liver adj3 (mortality or fibrosis or significant or poor or outcome)).ti,ab. |
| 34 or/27-33 |
| 35 26 and 34 |
| 36 exp risk factors/ |
| 37 precipitating factors/ |
| 38 prevalence/ |
| 39 incidence/ |
| 40 (risk* or prevalen* or inciden* or predict* or associat*).ti,ab. |
| 41 or/36-40 |
| 42 35 and 41 |
