# Supplementary material for: Metabolic risk factors and incident advanced liver disease in non-alcoholic fatty liver disease (NAFLD): A systematic review and meta-analysis of population-based observational studies
Source: PLoS Med. 2020 Apr 30;17(4):e1003100. doi: 10.1371/journal.pmed.1003100 (PMC7192386; doi:10.1371/journal.pmed.1003100)
Supplement: S3 Table — (DOCX) [file pmed.1003100.s005.docx]

S3 Table: Risk of Bias using the QUIPS tool [18] for assessing bias in prognostic studies

| Study ID | Study Participation | Study attrition | Prognostic factor measurement | Outcome measurement | Adjustment for other prognostic factors | Statistical analysis and reporting | Overall rating |
| --- | --- | --- | --- | --- | --- | --- | --- |
| *Alexander 2019* | low | low | low | low | low/moderate | low | ***LOW*** |
| *Andreasson 2017* | low | low | low | low | low/moderate | low | ***LOW*** |
| *Björkström 2019* | low | low | low | low | low/moderate | low | ***LOW*** |
| *El-Serag 2004* | low | low/moderate | low | low/moderate | moderate | moderate | ***MODERATE*** |
| *Goh 2017* | low | low | low | low | low/moderate | low/moderate | ***LOW*** |
| *Golabi 2018* | low/moderate | low/moderate | low | low | moderate | moderate | ***MODERATE*** |
| *Hagström 2016* | low | low/moderate | low | low | high/moderate | low | ***MODERATE*** |
| *Hagström 2018* | low | low | low | low | low/moderate | low | ***LOW*** |
| *Hagström 2019* | low | low | low | low | low/moderate | low | ***LOW*** |
| *Ioannou 2003* | low | low | low | low | low/moderate | low | ***LOW*** |
| *Ioannou 2005* | low | low | low | low | low/moderate | low/moderate | ***LOW*** |
| *Kanwal 2019* | low | low | low | low/moderate | Low/moderate | low | ***LOW*** |
| *Liu 2010* | low | low/moderate | low/moderate | low | low | low | ***LOW*** |
| *Nderitu 2017* | low | low | low | low | high/moderate | low/moderate | ***MODERATE*** |
| *Otgonsuren 2013* | low/moderate | low | low | low | low/moderate | low/moderate | ***LOW*** |
| *Pang 2018* | moderate | moderate | low/moderate | low | low/moderate | low/moderate | ***MODERATE*** |
| *Porepa 2010* | low | low | low/moderate | low | low/moderate | low | ***LOW*** |
| *Schult 2011* | low | low/moderate | low | low | moderate | moderate | ***MODERATE*** |
| *Schult 2018* | low | low | low/moderate | low | moderate | high/moderate | ***MODERATE*** |
| *Simeone 2017* | low | high/moderate | low | high/moderate | high | high/moderate | ***HIGH*** |
| *Stepanova 2010* | low | low | low | low | moderate | moderate | ***MODERATE*** |
| *Younossi 2013* | low | low | low | low | low/moderate | low/moderate | ***LOW*** |
