# Supplementary material for: Metabolic risk factors and incident advanced liver disease in non-alcoholic fatty liver disease (NAFLD): A systematic review and meta-analysis of population-based observational studies
Source: PLoS Med. 2020 Apr 30;17(4):e1003100. doi: 10.1371/journal.pmed.1003100 (PMC7192386; doi:10.1371/journal.pmed.1003100)
Supplement: Attachment — Submitted filename: Response to the editor reviewers_final.docx [file pmed.1003100.s006.docx]

20^th^ January 2020

Ref: PMEDICINE-D-19-04140

Dear Editor,

Thank you for considering the above manuscript for publication in PLOS Medicine and for the helpful comments from yourself and the reviewers.

We have included below the comments made by yourself and the reviewers in italics and, following these, our responses and the location of any changes made as a result of these comments. The line/page numbers refer to those on the marked up version (version with all tracking open to view) of the revised manuscript. We have also submitted a clean manuscript with these changes accepted.

**Requests from the editor:**

*Please provide p values in the abstract (and main text and tables) as well as removing funding information from the abstract.*

We have provided p values as available to abstract, text and tables 1 and 2.

Funding information has been removed from the abstract.

*Abstract and throughout – please remove causal language such as “This review demonstrates that T2DM leads to a greater than two fold increase in the risk of developing severe liver disease. “ Causality cannot be shown from systematic reviews.*

Removal of causal language: - line 78 changed to ‘is associated with’ rather than ‘leads to’ in the final paragraph of the abstract on page 3. ‘Increases the risk of’ has been changed to ‘is associated with’ on line 560 , second paragraph of the discussion on page 32.

*“Structured abstract” should simply read “Abstract”*

‘structured abstract’ has been changed to ‘abstract’ – line 45, page 2.

*Line 174 “Two researchers (HJ, GS or DS)” surely 3 researchers? Also line 183*

For clarity all screening and data extraction was done by HJ and ONE other (either DS or GS) – I have now made this clearer on lines 207, page 8 of the methods section.

*Line 354 – remove bold font, please*

Bold font removed from lines 380, page 23 of results section, and line 420, page 25 of results section.

*Please present references in square brackets (rather than superscript) at the end of each sentence, prior to the full stop.*

References have been changed to square bracket format throughout.

*Abstract*

*Please report your abstract according to PRISMA for abstracts, following the PLOS Medicine abstract structure (Background, Methods and Findings, Conclusions)*

The abstract is now presented in 4 short paragraphs with subtitles background, methods and findings and conclusions and follows the PRISMA guidelines for abstract structure.

*Abstract Background: Provide expand on the context of why the study is important.*

A sentence has been added to the abstract on lines 50/51 – page 2 which reads : “ **Finding people at high risk of experiencing these complications is important in order to prevent disease progression** “ to expand on the context of why the study is important.

*Abstract Methods and Findings: Please provide the dates of search, synthesis/appraisal methods and main outcome measure(s).*

The search date (updated to January 2020) is included (line 57, page2) as well as the synthesis, appraisal methods and main outcome measure.

*In the last sentence of the Abstract Methods and Findings section, please describe the main limitation(s) of the study's methodology.*

The main limitation of the study methodology has been added on lines 74-76, page 3 which reads: “ **Significant study heterogeneity observed in the meta-analyses was acknowledged to be a limitation, as was the potential effect of competing risks on outcome.”**

*Please begin your Abstract Conclusions with “In this study, we observed ..." or similar. Please avoid vague statements such as "this finding requires a health policy response", mentioning only specific implications substantiated by the results.*

The abstract conclusion has been amended to reflect these requests:

Lines 78-82, page 3 now reads:” **In this review, we observed that T2DM is associated with a greater than two fold increase in the risk of developing severe liver disease. As the incidence of diabetes and obesity continue to rise, using these findings to improve case finding for those at high risk of liver disease will allow for effective management to help address the increasing morbidity and mortality from liver disease”.**

*At this stage, we ask that you include a short, non-technical Author Summary of your research to make findings accessible to a wide audience that includes both scientists and non-scientists. The Author Summary should immediately follow the Abstract in your revised manuscript. This text is subject to editorial change and should be distinct from the scientific abstract*

Non-technical Author summary has been included on lines 87-107, pages 3-4.

*Please remove subheading ‘Main text’*

Main text subheading has been removed from line 108, page 4

*Introduction: If there has been a systematic review of the evidence related to your study (or you have conducted one), please refer to and reference that review and indicate whether it supports the need for your study.*

There has not to our knowledge been a systematic review on the evidence related to this study hence the need for this systematic review and meta-analysis

*Methods*

*When completing the PRISMA checklist, please use section and paragraph numbers, rather than page numbers*.

PRISMA checklist updated using section and paragraph numbers.

*Please update your search to the present time. We require that SRs are updated to within roughly 6 months of the expected publication date.*

Search has been updated to January 2020 with the addition of one further cohort study published in November 2019. The figures and tables have been updated to reflect the inclusion of this study (Kanwal et al [31]). As well as contributing to both the meta-analyses, this new study has some additional data on combined metabolic risk factors/metabolic syndrome and associations with severe liver outcomes. This is reflected in slight changes to the relevant sections in the narrative results as detailed below :

Lines 506-509, page 29 additional text has been added: **“A large population based data linkage study of over 270,000 (over 95% male) supports these findings with a reported HR of 1.23, CI (1.19-1.28), p<0.001 for an outcome of cirrhosis using a combined dyslipidaemia exposure based on low HDL and/or high triglycerides [31]”.**

Lines 517-520, page 30 text has been changed from discussion of 1 paper to 1 papers:

**“This is contradicted by two larger population based data-linkage studies looking at non-fatal severe liver disease, which both report a positive association between diagnosed hypertension and an incident liver outcome with HR 1.23, 95% CI 1.14-1.31, p<0.001) [36] and HR 1.59, 95% CI 1.51-1.69, p<0.001 [31]”.**

Lines 540 – 544, page 31 additional text has been added: **“A more recent, larger population data linkage study looked in more detail at combinations and numbers of metabolic risk factors associated with cirrhosis outcomes, reporting increasing HRs for those with increasing numbers of risk factors, with a HR of 2.56, 95% CI 2.26-2.92, p<0.001 for those with T2DM, obesity, hypertension and dyslipidaemia [31].”**

Lines 530 – 533 and line 537 page 31 – text has been removed as this sentence is no longer accurate considering the addition of new study and this also allows space for the additional text listed above without the paragraph becoming too wordy.

*Results*

*Line 144 - please define NCEP ATP*

Now defined on line 176

*Line 182 - please define CHARMS-PF*

Now defined on line 216

*Line 402 - please revise to ‘than’*

Now changed in line 477, page 29 from ‘that’ to ‘than’

*Figures 2 & 3 - When a p value is given, please specify the statistical test used to determine it (in the figure legend)*

I have added this information to the Fig 2 and 3 legend (it is also specified within the Figs). See lines 396-397, page 23 for Fig2 and lines 441-442, page 26 for Fig3

*Table 1 & 2 - please define all abbreviations in the table legend.*

Now included in table legends

See lines 336-349, page 18 for Table 1 legend

See lines 461-462, page 27 for Table 2 legend

*Discussion*

*Please present and organize the Discussion as follows: a short, clear summary of the article's findings;*

Presented in first 2 paragraphs of the discussion - lines 548-566, page 32

*what the study adds to existing research and where and why the results may differ from previous research;*

presented in paragraphs 3 – 7 of discussion - lines 567 – 607, page 32-34

*strengths and limitations of the study;*

paragraphs 8 and 9 of discussion – lines 609-627, page 34-35

*implications and next steps for research, clinical practice, and/or public policy;*

paragraphs 10 and 11 of discussion – lines 669-683, page 36-37

*one-paragraph conclusion.*

Last paragraph of the discussion – lines 684-689, page 37

*References*

*Please provide the names of the first 6 authors prior to ‘et al’ being used*

*Please ensure that journal titles are consistent e.g. Lancet/The Lancet*

References have been altered to this format and journal titles made consistent.

**Comments from the reviewers:**

*Reviewer #1: In this systematic review and meta-analysis, the authors analyze metabolic risk factors and their potential to predict liver disease outcomes in those with NAFLD and in the general population at risk for NAFLD. The authors find that type 2 diabetes leads to a greater than 2 fold increase in the risk of developing severe liver disease. It is well written, includes an extensive search for relevant studies, and with a large number of patients.*

*My comments are the following:*

*-	Although AASLD and NICE to don't recommend screening for NAFLD, would add that the American Diabetes Association most recent guidelines do recommend screening for fatty liver disease and advanced fibrosis in those with diabetes. This complements the findings of this study nicely.*

Thank you for pointing out this additional reference. I have added this in to the introduction as an organisation supportive of case finding in diabetes alongside the European Association for the Study of the Liver (EASL) and I agree this study adds to the evidence justifying their position on this.

-	*I am concerned about the inclusion criteria - there are only a few studies that have a specific diagnosis of NAFLD; the rest include only general populations at risk of NAFLD after excluding those with alcohol history and other causes of liver disease as the authors state in the methods and table 1. However, I reviewed one of the articles included in the meta-analysis (Andreasson 2017) and it does not appear that viral hepatitis or other types of liver disease were excluded. Can the authors comment on this?*

*This is important as underlying liver disease (i.e. hepatitis c) will have a compounding effect on those with diabetes to result in progressive / advanced liver disease. By not excluding other causes of liver disease, this no longer becomes a study about NAFLD but rather a general population study.*

Thank you for raising this. Decisions on the study inclusion criteria were pre-defined but due to the heterogeneity of the studies included, many had differing methods of excluding or adjusting for other causes of liver disease. In the Andreasson study [21] you mention, they excluded those with ‘severe liver disease’ at baseline based on ICD-coding (see first line of the results section in the paper) and also carried out a sensitivity analysis where they excluded those with a severe liver disease diagnosis within 2 years of cohort entry (which did not affect the estimates).

Although this study and some of the others do not explicitly report the aetiology of the liver diseases excluded we would expect that diagnosed viral hepatitis will have been coded and would appear in a list of ‘liver disease at baseline’. We have altered the text in the discussion to acknowledge more clearly that we have made this assumption, and that this is a study limitation, as well as why we feel our inclusion of these studies is justified.

Lines 74-76, page 3 of abstract, text added to acknowledge this as a study limitation:

“ **Significant study heterogeneity observed in the meta-analyses was acknowledged to be a limitation, as was the potential effect of competing risks on outcome”.**

Lines 610-616, page 34 of discussion, text added to further discuss this and acknowledge again as a study limitation:

**“In this synthesis we included data from population cohorts without a definite clinical diagnosis of NAFLD at baseline. It is therefore possible that not all liver outcomes in these groups were due to underlying NAFLD, which is a study limitation. All included studies reported that people with known liver disease of other common aetiologies (which would include viral hepatitis) were excluded and have adjusted for alcohol in the analysis. However, the possibility of other undiagnosed pathologies cannot be fully excluded.”**

Lines 616-622, page 34, text altered slightly to clarify justification and strengths of including this data:

**“ A recent multi-site European cohort study found that metabolic risk factors predicted cirrhosis with similar effect sizes for people with and without a coded diagnosis of NAFLD [20] and suggested this was likely due to NAFLD not being diagnosed or accurately coded (i.e. hidden disease in the control group). Other studies have also reported lower than expected levels of diagnostic coding for NAFLD [50]. This suggests that our approach may be a strength, as only a minority of people living with NAFLD have had a formal diagnosis, and so represent a highly selected sub-group.”**

*In addition, there are some studies that do not explicitly state that other liver disease was excluded (i.e. Liu 2010) - why was this?*

Thank you for raising this. We assume this is referring to the column in Table 1 ‘diagnosis of NAFLD at baseline’, where some of the cells with ‘no’ did not have ‘exclusion of other causes of liver disease’ or similar. This was an error in formatting the table and has been corrected to reflect that all included studies were required to have excluded/adjusted for other causes of liver disease as discussed above if they did not already have a diagnosis of NAFLD made as an inclusion criteria for the cohort.

See Table 1 – 5^th^ column from the left

-	*Also in table 1, the column with "? Diagnosis of NAFLD at baseline" is unclear - what is the question?*

We have changed this and it now reads ‘diagnosis of NAFLD at cohort inclusion’, to make it clearer as to what the ‘yes’ or ‘no’ refer to in this column.

See Table 1 – 5^th^ column from the left

-	*In table 1 - please include the reference for each study after author/year.*

This has now been included in Table 1 in the format [ref] after the author/year.

-	*While I understand the authors' point that only a minority of people living with NAFLD have had a formal diagnosis and that these patients maybe a highly selected group, I still find the title of the study misleading - instead of the NAFLD population, I would argue that this is a general population; yes, the majority of these patients likely have NAFLD, but without a linking diagnosis (through coding, liver tests, imaging), one cannot make that conclusion.*

Thank you for raising this point related to your previous concerns. The fact that this is a systematic review which includes unselected populations at risk of NAFLD, as well as general populations with a diagnosis of NAFLD is made clear throughout the text in the introduction, methods and discussion. In response to your concerns, we have more clearly acknowledged the possibility that non-NAFLD causes of liver disease may have contributed to the outcomes and cite this as a study limitation. (see quotes above from altered abstract and discussion to reflect this).

To change the title would, we feel, be confusing for the reader and prevent easy searching. The assumption made that the vast majority of liver outcomes in those with metabolic risk factors (after excluding/adjusting for alcohol and other liver diseases) are due to NAFLD is we feel a reasonable one. We are willing to adjust the title if it is felt to be misleading.

*Overall an excellent study with very strong public health implications.*

*Reviewer #2: Jarvis et al have performed a systematic review and meta-analysis on risk factors for development of severe liver disease, defined as fatal or non-fatal ICD-coding corresponding to cirrhosis or complications thereof. Main results are that primarily T2D is the major risk factor for severe liver disease, with obesity also a significant risk factor.*

*The review and analyses is appropriate, and gives somewhat more precise estimates than the individual studies. However, I think the paper could be strengthened, if possible, by adding sex-specific analyses (is the effect of T2D/obesity similar in men and women?) and by age categories?*

Thank you for these comments. Other than the studies that included only men [23,26,27,31,37] or women [28,32,38] , only one of the studies Andreasson [21] reported sex specific analyses, meaning it was not possible to provide any additional sex specific analysis beyond this. None of the included studies reported sub-group analysis by age, although the age range of the included cohort did vary between studies which is reported in Table 1. We agree that more detailed breakdown of risk of outcome by sex and age would be very interesting but was not possible with the study data published/reviewed.

*Also, if possible would be interesting to see effect of T2D + obesity. Could be added to Table 2.*

There were only a few studies that looked at the additive effect of metabolic risk factors, so this could not be analysed beyond the narrative discussion given. The new study added after the search update, Kanwal et al November 2019 [31], provides the most comprehensive look at different multiple risk factor combinations and is newly referenced in this narrative section.

Lines 541-545, page 31 text added: **“A more recent, larger population data linkage study looked in detail at combinations and numbers of metabolic risk factors associated with cirrhosis outcomes, reporting increasing HRs for those with increasing numbers of risk factors, with a HR of 2.56, 95% CI 2.26-2.92, p<0.001 for those with T2DM, obesity, hypertension and dyslipidaemia [31].”**

*Minor: Ref #46 seem to be an abstract from a conference. Please refer to the final paper.*

The published reference for this work has now been added – now reference [47] – line 921

*Reviewer #3: Alex McConnachie, Statistical Review*

*The paper by Jarvis et al presents the results of a systematic review and meta-analysis of incident advanced liver disease outcomes and metabolic risk factors. This review considers the use of statistics in the paper. Overall, these are very good, and my comments are quite minor.*

*The abstract could perhaps mention the high levels of heterogeneity observed in the meta-analyses.*

This is now mentioned as part of the abstract. See lines 74-75, page3. Text added: “ **Significant study heterogeneity observed in the meta-analyses was acknowledged to be a limitation”.**

*The confidence interval reported on line 438 (0.07; 0.01-.03) is clearly wrong.*

This was a typo – the upper limit has now been corrected to 0.3 rather than 0.03 (see line 518, page 30)

*I find it more useful for funnel plots to have the funnel added.*

We have added the 95%CI (funnels) to the funnel plots in the supplementary material (see S1 Fig and S2Fig revised versions)

Thank you again for these constructive comments which we hope we have comprehensively addressed.

Yours sincerely

Dr Helen Jarvis (on behalf of all the authors)
